# Successful Splenectomy for Hypersplenism in Wilson’s Disease: A Single Center Experience from China

**DOI:** 10.1371/journal.pone.0124569

**Published:** 2015-04-24

**Authors:** Liang-Yong Li, Wen-Ming Yang, Huai-Zhen Chen, Yun-Hu Wu, Xiang Fang, Jing Zhang, Zhen Wang, Yong-Sheng Han, Yu Wang

**Affiliations:** 1 Department of Neurology, the First Affiliated Hospital of Anhui University of Traditional Chinese Medicine, Hefei 230031, China; 2 Department of General Surgery, the First Affiliated Hospital of Anhui University of Traditional Chinese Medicine, Hefei 230031, China; 3 Institute of Neurology, Anhui University of Traditional Chinese Medicine, Hefei 230061, China; 4 Department of Neurology, the First Affiliated Hospital of Anhui Medical University, Hefei 230022, China; Texas A&M Health Science Center, UNITED STATES

## Abstract

Splenomegaly and pancytopenia are common in Wilson’s disease (WD) and splenectomy is one of the conventional treatments for splenomegaly and the associated pancytopenia. However, splenectomy remained controversial for hypersplenism in WD as it was reported that splenectomy leaded to serious emotional and neurological deterioration in WD patients with hypersplenism. In the current study, we present our experiences in 70 WD patients with hypersplenism who had undergone splenectomy, outlining the safety and efficacy of splenectomy in WD. The clinical database of 70 WD patients with hypersplenism who had undergone splenectomy in our hospital between 2009 and 2013 were reviewed and followed-up regularly. Before splenectomy, all the patients accepted a short period of anti-copper treatment with intravenous sodium 2, 3-dimercapto-1-propane sulfonate (DMPS). All the patients demonstrated a marked improvement in platelet and leucocyte counts after splenectomy. No severe postoperative complication was observed. In particular, none of the 37 patients with mixed neurologic and hepatic presentations experienced neurological deterioration after splenectomy, and none of the patients with only hepatic presentations newly developed neurological symptoms. During the one year follow-up period, no patient presented hepatic failure or hepatic encephalopathy, no hepatic patient newly developed neurological presentations, and only 3 patients with mixed neurologic and hepatic presentations suffered neurological deterioration and these 3 patients had poor compliance of anti-copper treatment. Quantative analysis of the neurological symptoms in the 37 patients using the Unified Wilson’s Disease Rating Scale (UWDRS) showed that the neurological symptoms were not changed in a short-term of one week after splenectomy but significantly improved in a long-term of one year after splenectomy. Additionally, compared to that before splenectomy, the esophageal gastric varices in most patients significantly improved one year after splenectomy. Thus, we may conclude that splenectomy is a safe and effective therapeutic measure for hypersplenism in WD patients who had been preoperatively treated with DMPS for powerful anti-copper therapy.

## Introduction

Wilson’s disease (WD) is an autosomal recessive disorder of copper metabolism which results in pathological accumulation of copper in various tissues and organs, predominantly in the liver, brain, and cornea. Toxic copper accumulation in these tissues and organs contribute to the neurological, psychiatric and hepatic symptoms including that of acute or chronic hepatitis, fulminant hepatic failure, and cirrhosis [1,2,3,4]. Cirrhosis and hypersplenism in WD patients are frequent and similar to that found in patients with cirrhosis of other aetiologies. Hypersplenism is clinically characterized by splenomegaly, thrombocytopenia, leukopenia, and anemia[5]. Thrombocytopenia and leucopenia increase the risk of spontaneous bleeding and bacterial infections. Furthermore, some anti-copper drugs, such as penicillamine, trientine and tetrathiomolybdate can occasionally cause bone marrow depression, anemia, leucopenia, immunological lesions, and even paradoxical symptom worsening[6,7,8]. Therefore, anti-copper therapy must often be stopped in patients with WD, which invariably leads to progression of the disease [2,4].

Liver transplantation is an effective option for hypersplenism in WD patients, but the organ shortage and high medical cost restrict its applicability [9,10,11,12]. Additionally, liver transplantation is most commonly indicated for WD patients with fulminant hepatic failure or decompensated liver cirrhosis unresponsive to medical therapy [10], and the emergence of neurological symptoms in WD was found significantly associated with poorer survival rate after liver transplantation [12]. Splenectomy is another option for hypersplenism and has been demonstrated to be able to reduce liver fibrosis and cause beneficial immunological changes in patients with cirrhosis caused by hepatitis [13]. However, it was reported that splenectomy for the treatment of hypersplenism leaded to serious emotional and neurological deterioration in WD patients [14]. Therefore, splenectomy remained controversial for hypersplenism in WD. In fact, the published literatures about the application of splenectomy for hypersplenism in WD are very scarce, thus the role of splenectomy for hypersplenism in WD remains suspended to be clarified.

In this study, we present our experiences of splenectomy for hypersplenism in 70 WD patients, outlining its safety and efficacy. To our knowledge, this is a summary from the largest sample collection of splenectomy for hypersplenism in WD patients.

## Methods

### Ethics statement

The protocol for this clinical study was in full compliance with the ethical principles of the Declaration of Helsinki and was consistent with the Good Clinical Practice guidelines and with applicable local regulatory requirements. Written informed consent was available from each adult patient or from the parents or guardians of the child patients. This study and the written consent procedure were approved by the Institutional Review Board of the First Affiliated Hospital of Anhui University of Traditional Chinese Medicine.

### Patients

In this study, 70 (46 males and 24 females) WD patients with hypersplenism were recruited from the Neurological Department, the first affiliated hospital of Anhui University of Traditional Chinese Medicine, in between 2009 and 2013. Mean age was 18.1 years (range, 9–42 years). All patients had been on chelation treatment with D-penicillamine, succimer, and zinc products, and the duration of chelation therapy ranged from 1 year to 22 years. Clinical manifestation was hepatic in 33 patients presenting signs of cirrhosis, without any neurological symptoms, and mixed hepatic and neurologic in 37 patients. The neurological presentations of the 37 WD patients included dysarthria, tremor, drooling, dystonia, incoordination, dysphagia, spasticity and psychiatric symptoms, etc. The hepatic function of the each patient was classified into three groups (class A, B and C) based on the Child-Turcotte-Pugh score (5 to 15-point scale), i.e. Child-Pugh A, B and C, which was originally created to evaluate the mortality in patients accepting surgery for portal hypertension [15], and is currently used to determine the survival of patients with chronic liver disease and cirrhosis. 50 WD patients with hepatic function categorized into Child-Pugh A and 20 patients with hepatic function categorized into Child-Pugh B were recruited into surgical processes, while the Child-Pugh C patients were not recruited into surgical processes. The endoscopic records and grading of esophageal varices was based on the criteria edited by the Digestive Endoscopy Branch of the Chinese Medical Association in 2000 [16]. The esophageal varices was graded into three degrees: Mild, the esophageal varices was strait or mildly tortuous; Moderate, the esophageal varices was tortuous and uplifted; and Severe, the esophageal varices was bead-like, nodular, or teardrop-like. Endoscopy before splenectomy revealed that the esophageal gastric varices was mild in 5 patients, moderate in 42 patients and severe in 23 patients. 6 patients had a history of upper gastrointestinal hemorrhage. Patients who had cirrhosis in combination with severe jaundice, refractory ascites, or other advanced liver disease, and those who had severe heart disease or renal failure were excluded from surgery. The demographic and clinical features of the WD patients with hypersplenism for splenectomy were summarized in table (Table 1).

**Table 1:** Demographic and clinical features of the WD patients.

Patients (n)	70
Average age (years)	18.1 (9–42)
Gender (M/F)	46/24
Child-Pugh class	
Child-Pugh A	50
Child-Pugh B	20
Clinical profile	
Hepatic	33
Mixed	37
Serum ceruloplasmin (g/L)	0.075 ± 0.034
24-hr urinary copper excretion (μg/24 h)	316.36 ± 31.55

The diagnosis of WD was based on the clinical characteristic manifestations, positive family history, low serum ceruloplasmin (<0.2 g/L), elevated 24-hour urinary copper excretion (>100 μg/24 h), elevated liver copper (>250 μg/g dry weight), presence of a K-F (Kayser-Fleischer) ring, elevated 24-hour urinary copper excretion following the administration of 2×500-mg does of D-penicillamine (>1600 μg/24 h) and magnetic resonance imaging (MRI) of brain [17,18], and met the WD diagnostic criteria formed at the 8^th^ International Meeting on Wilson disease [19]. Hypersplenism was defined as splenomegaly with thrombocytopenia, leukopenia, anemia and bone marrow aspiration revealed by biopsy. Thus, the hypersplenism was synthetically determined by clinical laboratory data, ultrasonography and computed tomography examinations. All patients entering the splenectomy processes had hypersplenism and moderate to severe degree of splenomegaly, and they had no or a small amount of ascites.

### Preoperative treatment

Sodium 2, 3-dimercapto-1-propane sulfonate (DMPS) is a water-soluble chelating agent that has been used to treat metal intoxication in the former Soviet Union since the 1960s and in Germany since 1978 [20]. In China, especially in our Wilson’s disease center, DMPS has been used for decoppering in WD patients for several decades [21], and it is demonstrated to have a more powerful decoppering capacity than the widely used D-penicillamine [22]. All patients received strict, formal and powerful anti-copper treatment with a combination of zinc and intravenous DMPS at a dosage of 5–15 mg/kg per day for 6–8 weeks before splenectomy. During the treatment with combination of zinc and intravenous DMPS, 3 patients presented gingival bleeding, epistaxis, and transient mild decline of neutrophil and platelet, with or without nausea and vomiting, and those reactions disappeared after the dose of DMPS was decreased. Patients allergic to DMPS presenting skin rash, itching and fever, were excluded from this study. Meanwhile, all the patients should take a low copper diet. Patients with ascites were given low salt diet, diuretics and intravenous infusion of human albumin. Fresh frozen plasma was administered preoperatively to improve prolonged prothrombin time. Patients with platelet count less than 3.0×10^4^ /μL were treated with platelet transfusions and vitamins K1 one week before splenectomy. Meanwhile, all patients received systemic prophylaxis with intravenous antibiotics.

### Operative procedures

Patients received general anesthesia by endotracheal intubation combined with intravenous anesthesia. “L” incision in the left upper abdomen was employed in the surgery, and the splenic artery and vein were dissociated and ligated at the upper border of the pancreas. The splenectomy was performed routinely. Portaazygous devascularization was performed for all the patients with a history of upper gastrointestinal hemorrhage.

### Postoperative treatment

Standard postoperative care and treatment including intravenous antibiotics and adapted analgesic were given for all patients. Platelet transfusions, vitamins K1, frozen plasma and hemostatic agents were administered for patients with bleeding or bleeding tendency, and patients with ascites were treated with diuretics and albumin infusion. To actively improve the general condition of the patients, nutrition strengthening, electrolyte balance maintaining and secondary infection preventing were conducted regularly. Preventive tension-relieving suture was employed to avoid incision dehiscence and the stitches were taken out 14 days after splenectomy. Once the patients’ symptoms were stable, early and life-long regular anti-copper therapy was administered.

### Analytical assessment

Blood cell counts of leucocyte or white blood cell (WBC) and platelet (PLT) were monitored before splenectomy and by one day, one week, two weeks, three weeks, four weeks and one year after splenectomy. Liver function represented with aspartate aminotransferase (AST), alanine aminotransferase (ALT), total bilirubin (TBIL), and direct bilirubin (DBIL) in serum were measured before splenectomy and by one day, one week, one month and one year after splenectomy. To access the changes of neurological symptoms, the Unified Wilson’s Disease Rating Scale (UWDRS) [23] was used for each patient before splenectomy and by 1 day, 1 week, 1 year after splenectomy. The UWDRS had been demonstrated to be an appropriate tool to monitor disease progression and drug efficacy with internal consistency, interrater reliability and validity in WD patients [24], and it has now been well utilized in clinical assessment of WD [25,26]. This scale contains 3 sections including: consciousness, Barthel index, and neurological examination. A total score equal to 0 indicates the absence of neurological signs, and a total score ranging from 1 to 185 indicates the different severities of neurological impairment. B-type ultrasonic of spleen was repeated routinely after splenectomy. Meanwhile, the frequency and type of complications associated with splenectomy were recorded. All the patients were followed-up for 1 year.

### Statistical analysis

Statistical analyses were performed using a commercial statistical software package (SPSS for windows, version 13.0). Data were expressed as the mean ± SD. Paired *t*-tests were used for statistical analysis. Differences were considered significant at *P* < 0.05.

## Results

### Effects of DMPS therapy and splenectomy on serum copper concentration

The powerful anti-copper treatment with DPMS which lasted for 6 to 8 weeks obviously decreased the serum copper for each patient before splenectomy. Statistical analysis showed that this short period of powerful decoppering therapy significantly decreased the serum copper before splenectomy (Table 2). One year after splenectomy, the serum copper rised to level significantly higher than that in between post-DMPS therapy and pre-splenectomy but still significantly lower than that before DMPS therapy (Table 2).

**Table 2:** Serum copper concentrations before and after DMPS therapy and 1 year after splenectomy (mean±SD).

Time	Serum copper (μmol/L)
Before DMPS therapy	3.51±1.87
After DMPS therapy	1.75±0.69**
1 year after splenectomy	2.67±1.01* ^**^**^

**P < 0.01, compared to that before DMPS therapy

*P < 0.05, compared to that before DMPS therapy

^**^**^ *P* < 0.05, compared to that after DMPS therapy.

### Effects of splenectomy on blood cell counts

The WBC and PLT counts in WD patients with hypersplenism were obviously lower than their normal ranges (WBC count, 4–10×10^9^; PLT, 100–300×10^9^) (Table 3). The WBC counts were sharply elevated 1 day after splenectomy, then significantly decreased by 7 and 14 days after splenectomy, and stayed in normal range by 21, 28 days and 1 year after splenectomy. Though there was a significant decrease in WBC counts during the follow-up period of one week to one year after splenectomy as compared to those 1 day after splenectomy, the WBC counts during the follow-up period of one week to one year after splenectomy were significantly higher than those before splenectomy and remained in a normal range (Table 3). The PLT counts presented marked increase after splenectomy, reached its peak by 14 days after splenectomy and were then gradually decreased during the follow-up period of one week to one year after splenectomy (Table 3).

**Table 3:** Comparisons of WBC and PLT counts before and after splenectomy (mean±SD).

Variables	Pre-splenectomy	Post- splenectomy					
	1 day	1 day	1 week	2 weeks	3 weeks	4 weeks	1 year
WBC (×10^9^/L)	2.33±0.82	17.96±5.49*	8.73±3.18* ^	4.57±1.11* ^	5.10±1.13* ^	5.58±1.18* ^	6.24±1.28* ^
PLT (×10^9^/L)	47.34±34.13	107.78±40.07*	373.26±122.92* ^	463.20±114.01* ^	347.37±66.42* ^	340.88±71.63* ^	235.61±52.20* ^

WBC, white blood cell; PLT, platelet.

*p<0.01, compared to that pre-splenectomy

^p<0.01, compared to that 1 day post-splenectomy.

### Effects of splenectomy on liver function

The serum concentrations of ALT, AST, TB and DB in WD patients with hypersplenism were obviously higher than their normal ranges (ALT, 5–35 U/L; AST, 8–40 U/L; TB, 2–23.4 μmol/L; and DB, 0–9.8 μmol/L) (Table 4). The four markers for liver function were all sharply up-regulated 1 day after splenectomy, then significantly decreased by 1 week after splenectomy, and even, by one month after splenectomy, decreased to a level significantly lower that that before splenectomy (Table 4). By one year after splenectomy, the serum levels of ALT, AST, TB and DB were further decreased (Table 4).

**Table 4:** Comparisons of liver function before and after splenectomy (mean±SD).

Liver function	Pre-splenectomy	Post-splenectomy			
	1 day	1 day	1 week	1 month	1 year
ALT (U/L)	33.23±16.23	62.08±47.70*	30.90±14.61^	22.64±10.73* ^	19.77±9.93* ^
AST (U/L)	31.48±11.99	64.48±34.31*	30.03±12.29^	23.14±9.41* ^	21.38±8.67* ^
TB (μmol/L)	19.89±10.73	31.75±13.55*	18.95±9.91^	15.60±8.05* ^	15.07±8.09* ^
DB (μmol/L)	8.00±5.70	14.61±7.47*	8.01±5.95^	6.14±3.79* ^	5.85±3.67* ^

ALT, alanine aminotransferase; AST, aspartate aminotransferase; TB, total bilirubin, DB, direct bilirubin.

*p<0.01, compared to that pre-splenectomy

^p<0.01, compared to that 1 day post-splenectomy.

### Effects of splenectomy on neurological symptoms

37 patients with mixed hepatic and neurologic symptoms experienced no deterioration of neurological symptoms after splenectomy and during the one-year follow-up, and 9 out of the 37 patients had the neurological symptoms improved one year after splenectomy. 3 out of the 37 patients suffered deterioration of neurological symptoms including tremor, drooling and dysarthria in the one year follow-up period, and they were found have poor compliance of anti-copper treatment. Among the 33 patients whose main manifestation was hepatic, no patient developed neurologic symptoms after splenectomy and after the one-year follow-up. Quantative analysis of the neurological symptoms in the 37 patients using UWDRS showed that the neurological symptoms were not changed one week after splenectomy but significantly improved one year after splenectomy (Table 5).

**Table 5:** Neurological symptom scoring before and after splenectomy (mean±SD).

Time	Unified Wilson's Disease Rating Scale
1 day before splenectomy	19.74±6.33
1 week after splenectomy	20.18±6.61
1 year after splenectomy	16.41±5.89* ^

*P < 0.05, compared to that 1 day before splenectomy

^p<0.01, compared to that 1 week after splenectomy.

### Side effects and complications of splenectomy

All the patients experienced fever and incision pain during the hospitalization. Fever up to 38.5°C was noticed in 13 patients and lasted for 4–7 days. Transient ascites occurred in 21 patients. 18 patients developed portal vein thrombosis. However, all these postoperative complications were treated conservatively with a good outcome. Other severe postoperative complications such as bleeding, sepsis or other overwhelming symptoms were not observed. During the one year follow-up period, no patient presented hepatic failure or hepatic encephalopathy, 15 patients reported they had 1 to 3 times of mild to moderate fever probably due to common cold and none of them accepted antibiotic treatment and recovered well in days. Only one patient experienced severe pulmonary infection and was re-hospitalized, and this patient recovered well after antibiotic treatment for two weeks. Endoscopy after the one-year follow-up showed that the esophageal gastric varices was mild in 31 patients, moderate in 26 patients and severe in 4 patients. And no patient experienced gastrointestinal hemorrhage or re-bleeding. Compared to that before splenectomy, the esophageal gastric varices in most patients significantly improved one year after splenectomy. 9 patients refused re-examinations of endoscopy after splenectomy.

## Discussion

In our study, splenectomy successfully improved WBC and PLT counts and liver function. Additionally, we did not observe any instant severe complication. Part of patients experienced acceptable minor complications which were tolerable for the patients themselves and were treated with good outcomes. In particular, no deterioration of neurological symptoms was observed after splenectomy. During the one year follow-up period, neither dropping of WBC and PLT counts nor worsening of hepatic function occured. Only 3 patients suffered from neurological deterioration due to poor compliance of anti-copper treatment, all other patients had the neurological symptoms improved during the one year follow-up. Before the surgery of splenectomy, all patients accepted short period (6 to 8 weeks) of powerful decoppering therapy with DMPS and zinc.

Splenectomy has been used as a measure to treat cytopenia in patients with hypersplenism associated with many conditions, such as hematopoietic stem cell transplantation (HSCT) and Hepatitis C virus (HCV)-related liver cirrhosis [27,28]. Cytopenia and hypersplenism are common in WD patients with liver cirrhosis [5], whereas there are very few literature reports indicating the usage of splenectomy in WD [14,29,30]. In the current study, we futher confirmed the efficacy of splenectomy in the improvement of cytopenia associted with hypersplenism in WD patients with liver cirrhosis. Recently, literatures have shown that splenectomy can improve liver function in patients with liver cirrhosis caused by hepatitis C or by advanced hepatocellular carcinoma [31,32,33]. Here, we also show the long-term beneficial effect of splenectomy on liver function in WD patients with hypersplenism and liver cirrhosis, though the deterioration of liver function was observed shortly after splenectomy. It is quite unclear how the splenectomy results in an improvement of the liver function in WD patients. Studies in patients with hepatitis C virus-related liver cirrhosis and in animals with liver cirrhosis showed that splenectomy can attenuate liver fibrosis and inflammatory responses [13,34]. Other effects of splenectomy on liver have also been observed in rats with liver transplantation, such as suppressed hepatocellular apoptosis and increased hepatic regeneration probably through preventing excessive portal vein hepatic inflow and eliminating splenic inflammatory cell recruitment into the liver [35]. Thus the splenectomy-induced improving effcet on liver function in WD patients might be associated with its effects of anti-fibrosis, beneficial immunological changes and liver arterial blood supply improvement. On the other hand, the copper metabolism alterations after splenectomy might also contribute to the splenectomy-induced improving effcet on liver function in WD patients, as removal of the spleen in rats is followed by an increased elimination of copper in the feces [36]. Lastly, splenectomy may exert its liver function improvement effect in WD patients through elevating the copper-chelating rate, as it is found that a greater degree of iron overload in splenectomized patients results in improvement of deferiprone-chelated iron, as well as a significant increase in urinary iron excretion in β-thalassaemia patients [37], though it is the alteration of iron but not copper metabolism. This last speculation is seemly supported by our findings that the serum copper level one year after splenectomy was significant lower than that before intravenous DMPS treatment, though the chelating therapy had moved back to the D-penicillamine and zinc. Whereas, the level of serum copper increased 1 year after splenectomy in comparision with that shortly after DMPS therapy, though the dose of D-penicillamine was increased after splenectomy. This might be due to the less powerful anticoppering capacity of D-penicillamine in comparison to that of intravenous DMPS, and on the other hand, some patients may not strictly take a low copper diet.

Though hypersplenism and cytopenia are not uncommon in WD, up to now, there are very few literature reports indicating the usage of splenectomy for hypersplenism in WD patients, and all these few reports were published in the last century [14,29,30]. The lack of further reports of splenectomy in WD with cirrhosis and hypersplenism might be due to the deterioration of neurological symptoms evoked by splenectomy as reported in this early literature [14]. In fact, the deterioation of neurological symptoms in WD patients is more commenly observed at the initial stage of anticoppering treatment with different drugs [38,39,40,41]. We further observed that pre-symptomatic WD patients developed acute neurological symptoms and neurologically symptomatic WD patients suffered neurological deterioration after trauma, especially after traumatic brain injury (unpublished). The reason for this deterioration is uncertain. One theory explaining this neurological deterioration phenomenon involves the mobilization and redistribution of hepatic copper [2,39]. The copper is initially accumulatesd in the liver and then mobilizes to the blood, brain and other tissues, and the copper transported into the brain was mainly achieved through the blood-brain barrier (BBB) [42,43]. It was reported that the brain MRI abnormalities increased in WD patients after anti-copper drugs induced neurological deterioration [38,39,44]. Based on the blood-brain barrier (BBB) study in WD patients, Stuerenburg proposed that the increase in BBB disturbance enables mobilization of copper from the liver to the brain more readily, causing the deterioration of neurological symptoms during anti-copper treatment [45]. Our study showed that splenectomy caused no short-term or long-term deterioration but long-term improvement of neurological symptoms in WD patients with cirrhosis and hypersplenism. And all our patients received strict powerful decoppering therapy with intravenous DMPS, one of the best intravenous copper chelating agents with low toxicity and minor side effects [21,22] before splenectomy. Thus, we may speculate that the powerful decoppering therapy with DMPS, which significantly lowered the pre-operation serum copper concentration, may reduce the redistribution of serum copper to the CNS, thus causing no deterioration of neurological symptoms in short-term period after splenectomy in our study. And, in the long term period of the one year follow-up, improved neurological symptoms were observed after splenectomy. This might be due to the elevated copper-chelating rate caused by splenectomy similar to the elevated iron-chelating rate caused by splenectomy [37,46], as the serum copper level was sinificantly lower than that before the intravenous DMPS treatment, though the chelating therapy had moved back to the D-penicillamine and zinc.

Surgical wounds, general anesthesia and postoperative infection may exacerbate the existing liver damage in WD patients, enhancing the potential risk of hepatic encephalopathy and even of the life-threatening liver failure [14,47,48]. Thus, patient selection for surgery might be critical for the prognosis of splenectomy. In combination with our long previous experiences, the indications for splenectomy were in those patients with the following conditions: moderate to severe degree of splenomegaly; no or a small amount of ascites; both leukocyte and platelet or single decreasing, especially platelet count below 60×10^9^ /L and leukocyte count below 3.0×10^9^ /L; recurrence of digestive bleeding; liver function of Child-Pugh A and B. While the conditions of dramatic decreasing of platelet, severe hepatic injury and poor general condition intolerable for surgery were contraindications for splenectomy. On the other hand, once the patient has indications for splenectomy, splenectomy should be performed early. Otherwise, WD patients with hypersplenism can not tolerate the surgery due to the poor general condition and the occurrence of decompensated cirrhosis.

During the surgical procedure of splenectomy, gastrointestinal bleeding may occur in WD patients with hypersplenism. Devascularization but not a shunt was an ideal management for this condition, as a shunt may enable the copper in systemic circulation directly to the central nervous system, which may lead to emotional and neurological deterioration. Although laparoscopic splenectomy with minimally invasive has been widely used for hypersplenism in cirrhosis of other aetiologies [49,50], the presence of a large spleen and poor coagulation with unexpected hemorrhage in WD patients with hypersplenism is relative restriction for laparoscopic splenectomy [51]. On the other hand, our past experiences indicated that the selection of anesthesia technique and anesthetics was also important, as many anesthetics which are metabolized in the liver may be toxic to the liver [47,48]. In our study, we also observed the impairment of liver function after operation with the levels of AST, ALT, TBIL and DBIL temporarily increased 1 day after splenectomy. Thereby, we should choose anesthetics with the least hepatic toxicity and patients of hypersplenism in WD with severe hepatic injury should be excluded from this surgery to avoid the possible occurrence of hepatic failure during splenectomy. The technique of anesthesia should also be specific to individual patient [47]. Rigid dystonia in some WD patients may result in elevated abdominal muscle tone or abdominal muscular spasm[2], for these patients, endotracheal intubation combined with intravenous anesthesia was used to relax elevated abdominal muscle tone or abdominal muscular spasm during splenectomy. Meanwhile, preventive tension-relieving suture was utilized to avoid incision dehiscence due to elevated abdominal muscle tone after splenectomy. Finally, all patients accepting splenectomy should receive systemic prophylaxis with intravenous antibiotics to prevent secondary infection, and none of our patients experienced a serious postoperative infection.

## Conclusions

Splenectomy effectively improved the platelet and leucocyte counts and liver function in WD patients with hypersplenism. With the preoperative powerful decoppering therapy and good perioperative management, no severe postoperative complications were observed. In particular, no patient with mixed neurological and hepatic presentations experienced neurological deterioration and no patient with only hepatic presentations newly developed neurological symptoms after splenectomy. Our study confirmed the safety and efficacy of splenectomy for hypersplenism in WD patients. Longer time follow-up and larger sample size of cases would be useful to further estimate the clinical value of splenectomy for the management of hypersplenism in WD patients.
